# Silence of the Lambs: The Immunological and Molecular Mechanisms of COVID-19 in Children in Comparison with Adults

**DOI:** 10.3390/microorganisms9020330

**Published:** 2021-02-07

**Authors:** Francesca Cusenza, Giusy Davino, Tiziana D’Alvano, Alberto Argentiero, Valentina Fainardi, Giovanna Pisi, Nicola Principi, Susanna Esposito

**Affiliations:** 1 Pediatric Clinic, Pietro Barilla Children’s Hospital, University of Parma, 43126 Parma, Italy; francescacusenza7@gmail.com (F.C.); jewsee@hotmail.it (G.D.); tiziana.dalvano@gmail.com (T.D.); alberto.argentiero@unipr.it (A.A.); valentina.fainardi@gmail.com (V.F.); gpisi@ao.pr.it (G.P.); 2 Università degli Studi di Milano, 20122 Milan, Italy; nicola.principi@unimi.it

**Keywords:** children, COVID-19, immune response, MIS-C, SARS-CoV-2

## Abstract

Children infected by severe acute respiratory syndrome coronavirus 2 (SARS-CoV-2) can suffer from severe coronavirus disease 2019 (COVID-19). However, compared to adults and the elderly, susceptibility to SARS-CoV-2 infection in children seems to be lower; when infection does develop, most infected children remain asymptomatic or develop a mild disease. Understanding why children seem generally protected from severe COVID-19 and only rarely develop clinical conditions that can cause hospitalization, admission to the pediatric intensive care unit and death can be important. More details on the mechanism of action of SARS-CoV-2 could be defined. Moreover, the role played by children in virus diffusion should be better analyzed, and the development of effective preventive and therapeutic measures against COVID-19 could be favored. The main aim of this paper is to discuss the present knowledge on immunological and molecular mechanisms that could explain differences in COVID-19 clinical manifestations between children and adults. Literature analysis showed that although most children are clearly protected from the development of severe COVID-19, the reasons for this peculiarity are not fully understood. Developmental variations in immune system function together with the potential role of repeated antigen stimulation in the first periods of life on innate immunity are widely studied. As the few children who develop the most severe form of pediatric COVID-19 have certain alterations in the immune system response to SARS-CoV-2 infection, studies about the relationships between SARS-CoV-2 and the immune system of the host are essential to understand the reasons for the age-related differences in the severity of COVID-19.

## 1. Introduction

Children infected by severe acute respiratory syndrome coronavirus 2 (SARS-CoV-2) can suffer from severe coronavirus disease 2019 (COVID-19), as clearly shown by the reported cases of multisystem inflammatory syndrome in children (MIS-C) [1]. However, compared to adults and the elderly, susceptibility to SARS-CoV-2 infection in children seems to be lower; when infection does develop, most infected children remain asymptomatic or develop a mild disease [2]. A joint report from the American Academy of Pediatrics and the Children’s Hospital Association updated through 3 December 2020 showed that, in the USA, the cumulative number of pediatric COVID-19 cases was 1,460,905, a value that represents only 12.0% of all cases diagnosed in the country. In the USA, child hospitalization rates for COVID-19 were only 1.1–3.0% of total reported hospitalizations, while the child mortality rate was 0.00–0.23% of all COVID-19 deaths [3].

Although currently available epidemiologic data likely underestimates the real incidence of SARS-CoV-2 infections in children, as diagnostic testing focused on symptomatic individuals and hospitalized patients almost completely excluded asymptomatic children, there is no doubt that the risk of severe COVID-19 increases with age. In the USA, the lowest risk of death was found in children compared to subjects aged 18–29 years, who had a risk several times higher than children; those aged 40–49 years, 50–64 years, 65–74 years, 75–84 years and >85 years had risks of COVID-19-related death of 10, 30, 90, 220 and 630 times higher, respectively [4].

Understanding why children seem generally protected from severe COVID-19 and only rarely develop clinical conditions that can cause hospitalization, admission to the pediatric intensive care unit (PICU) and death can be important. More details on the mechanism of action of SARS-CoV-2 could be defined. Moreover, the role played by children in virus diffusion should be better analyzed, and the development of effective preventive and therapeutic measures against COVID-19 could be favored. The main aim of this paper is to discuss the present knowledge on immunological and molecular mechanisms that could explain differences in COVID-19 clinical manifestations between children and adults.

## 2. SARS-CoV-2 and Infection Development

SARS-CoV-2 uses angiotensin converting enzyme 2 (ACE2) to infect cells [5]. ACE2 is an important regulator of homeostasis of the renin–angiotensin system by degrading angiotensin II, which has proinflammatory, pro-fibrotic and vasoconstrictor activities, into angiotensin 1–7, which modulates vasoconstriction, fibrinogen activation and inflammatory cytokine expression [6]. The receptor is widely expressed in several human tissues, including cells of the upper and lower respiratory tract, heart, kidney, small intestine and, to a lesser extent, lung. Lung ACE2 expression is concentrated mainly in type II alveolar cells and macrophages and modestly in bronchial and tracheal epithelial cells. To infect cells, the transmembrane spike glycoprotein (S protein) of SARS-CoV-2 binds to the cellular membrane ACE2. Thereafter, a serine protease, TMPRSS2, cleaves the SARS-CoV-2 S protein, allowing for cell membrane fusion and endocytosis of the virus and subsequent viral replication [7]. Similar to what has been shown for other coronaviruses, such as SARS-CoV and human coronavirus NL63, SARS-CoV-2 downregulates ACE2 expression. This increases angiotensin 2 concentrations and imbalances the angiotensin 2/angiotensin 1–7 ratio. Additionally, SARS-CoV-2, when entering the cell, induces endocytosis and cleavage and then reduces the cell surface levels of the ACE2 receptor [8].

However, how the opposite effects of SARS on ACE2 receptor expression and angiotensin 2 metabolism impact COVID-19 severity is not clearly established. Reduced ACE2 expression could lead to a lower risk of severe disease due to reduced virus entry into cells. Neonatal sheep have lower ACE2 expression than adult sheep [9]. In humans, it has been shown that ACE2 gene expression was lowest (mean log2 counts per million, 2.40; 95% confidence interval (CI), 2.07–2.72) in children aged less than 10 years and tended to progressively increase in adolescents and young adults (mean log2 counts per million of 2.77 (95% CI, 2.64–2.90) for children aged 10–17 years, 3.02 (95% CI, 2.78–3.26) for young adults and 3.09 (95% CI, 2.83–3.35) for adults >25 years) [10]. Consequently, the reduced ACE2 expression in the first periods of life could explain the lower number of severe COVID-19 cases in children compared to adults and the elderly. However, several factors indicate that this cannot be the only explanation for the lower risk of severe COVID-19 in children. In animal studies, ACE2 has been found to be protective against SARS-CoV-2 lung damage [11]. Beyond adulthood, ACE2 expression tends to decrease considerably, which contrasts with the greater severity of COVID-19 among older people [12]. Finally, in patients with some underlying diseases, such as diabetes mellitus, who have reduced ACE2 expression, the risk of severe disease is significantly increased [13].

On the other hand, downregulation of ACE2 due to SARS-CoV-2 activity leading to angiotensin 2 accumulation can be deleterious. Increased angiotensin 2 concentrations are associated with vascular permeability, inflammation and tissue damage. As already shown in respiratory syncytial virus (RSV) [14] and avian influenza pneumonia [15], in adult patients with COVID-19, angiotensin 2 levels have significant positive correlations with SARS-CoV-2 viral load and the severity of respiratory impairment [16].

## 3. Immune Response to SARS-CoV-2 Infection

### 3.1. Innate Immune System

RNA viruses are recognized and bound by pattern recognition receptors (PRRs), such as toll-like receptor (TLR)-3 and TLR-7, which are expressed on the endoplasmic reticulum membrane of macrophages, dendritic cells and B lymphocytes, and by RIG-I and MDA5, which are located in the cytoplasm. This activates the transcription factors NF-κB and interferon regulatory factor 3 (IRF3) and causes increased expression of innate proinflammatory cytokines (interleukin (IL)-1, IL-6 and tumor necrosis factor (TNF)-α) and type 1 interferon (IFN) [17]. Similar to SARS-CoV and Middle East respiratory syndrome (MERS)-CoV, SARS-CoV-2 can interfere with this pathway through its M, N, NSP1 and PLpro proteins, altering ubiquitination and degradation of the RNA sensor adaptor molecules MAVS and TRAF3/6 and attenuating type 1 IFN production [18,19]. Antagonism of type 1 IFN production aids viral replication, resulting in increased release of pyroptosis products and an aberrant inflammatory response [20]. Pyroptosis is a highly inflammatory form of programmed cell death that is commonly seen with intracellular pathogens and is part of the antimicrobial reaction [21]; it is mediated by the production of IL-1β, an important cytokine released during pyroptosis and upregulated during SARS-CoV-2 infection [22].

Channappanavar et al. [23] demonstrated that, while an effective innate immune response during SARS-CoV infection leads to mild–moderate disease, with early type 1 IFN production and a reduced virus titer, severe disease results from delayed expression of type 1 IFN and an inappropriate inflammatory response, with a high virus titer, increased monocyte-macrophage and neutrophil accumulation in the lungs and massive production of proinflammatory cytokines (IL-1, IL-6, IL-8, CXCL-10 and MCP-1).

The massive release of cytokines causes cytokine storm syndrome, a form of systemic inflammatory response syndrome. The complex formed by IL-6 and its receptor IL-6Rα is the main in vivo stimulator of the transcription factor STAT3 during inflammation [24].

STAT3 is necessary for the full activation of NF-κB, and both are able to activate the IL-6 amplifier (IL-6AMP), causing the production of IL-6 with a positive feedback mechanism. This in turn leads to greater activation of STAT3 and NF-κB through a chain reaction. IL-6AMP also induces other proinflammatory cytokines and is able to recruit lymphoid and myeloid cells [25].

The milder clinical presentation of COVID-19 in children could be explained by reduced serum levels of cytokines and a different basal inflammatory state. It has been shown that cytokine production increases with age, a widely studied phenomenon named “inflamm-ageing”, which is responsible for a subclinical inflammatory state predisposing patients to the development of various inflammatory diseases (i.e., cancer, cardiovascular diseases, diabetes, osteoporosis and dementia) [26,27,28]. Indeed, previous studies have shown an age-associated increase in the production of various cytokines, such as IL-1β, IL-2, IL-4, IL-6, IL-10, IFN-γ and TNF-α, a characteristic that would be advantageous in children, as they would therefore be less likely to develop cytokine storm syndrome [29,30]. Furthermore, even in children with a severe clinical condition, the levels of inflammatory mediators are reduced, with a better prognosis. Schouten et al. [31] compared 49 children with acute respiratory distress syndrome (ARDS) to 43 adults and showed significantly lower levels of inflammatory biomarkers (MPO, IL-6 and IL-10) in the bronchoalveolar lavage (BAL) fluid in children and no deaths registered in this group.

The reduced risk of severe COVID-19 in children could also be due to differences in natural killer (NK) cell number and activity. NK cells are a part of innate immunity and are known to eliminate virally infected cells [32]. Healthy children have a greater number of NK cells than adults and the elderly [33], which could explain the better response of children to SARS-CoV-2 infection, although the ability of NK cells to produce cytokines does not exclude that they may play a role as pathogenic factors. However, recent studies have shown that, in adults with severe COVID-19, together with a reduced number of total NK cells [34], an increased number of activated cytokine-producing CD56 bright NK cells could be demonstrated [35]. The hyperinflammation systematically found in severe COVID-19 may depend, at least in part, on a SARS-CoV-2-induced mechanism of NK activation. Although mechanisms conditioning the different NK functions are unknown, differences between children and adults in activation processes may play a role in favoring the lower severity of pediatric COVID-19.

### 3.2. Adaptive Immune System

Adaptive immunity is based on the activity of lymphocytes, such as CD4 helper T cells, CD8 cytotoxic T cells and B cells. CD8 T cells are important for directly attacking and killing cells infected by intracellular pathogens, whereas B cells secrete antibodies, and CD4 T cells induce cytokine production and prime both CD8 T cells and B cells [28].

In SARS-CoV-2 infection, a delayed and ineffective innate immune response leads to a dysregulated adaptive response, aggravating inflammation [36]. Proinflammatory cytokines activate Th1-type cells and B cells, with an expansion of CD4 and CD8 T cells and a reduction in regulatory T cells. Regulatory T cells contribute to the homeostasis of the immune system, suppressing the excessive activation and proinflammatory activity of most lymphocytes. In patients infected by SARS-CoV-2 with severe disease, the blood levels of CD4, CD8 and regulatory T cells are remarkably lower than those in patients with mild–moderate disease, resulting in a decreased lymphocyte count and lymphocytopenia [20]. The first autopsy of a patient who died due to SARS-CoV-2 infection showed an accumulation of mononuclear cells, particularly monocytes-macrophages and T cells, in the lungs, suggesting that T cells migrate away from the blood to these sites to control the infection contributing to the depletion of blood lymphocytes [20]. On the other hand, in children with SARS-CoV-2 infection, lymphocyte levels are steadily in the normal range, suggesting less immune dysregulation than in adults [37]. Various hypotheses could explain this characteristic. Lymphocyte levels are physiologically higher in children than in adults, consisting mainly of NK and CD8 cells [31]. Moreover, in children, most naive T cells develop in the thymus, which is larger in childhood than in adulthood and undergoes involution with aging, while naive T cells self-renew exclusively in the peripheral blood in adults and elderly individuals [38]. Naive T cells of adults and elderly individuals present a restricted T cell receptor repertoire, with consequently defective expansion and differentiation into effector cells compared to those of the naive T cells of children and a decreased response against new antigens [39].

## 4. Previously Developed Immunity against Coronaviruses

In children, infections due to human CoVs (HCoVs) other than SARS-CoV and SARS-CoV-2, such as HCoV-NL63, HCoV-HKU1, HCoV-OC43 and HCoV-229E, are extremely common [40]. Studies have shown that some SARS-CoV-2 spike peptides have high homology to those of common CoVs; therefore, it has been supposed that cross-reactive pre-existing neutralizing antibodies and long-lasting T cell immunity against these viruses could protect children from SARS-CoV-2 infection and explain their lower hospitalization and death rates compared to adults during the COVID-19 pandemic [41]. However, several findings oppose this hypothesis. Animal and human studies have shown that most of the cross-reacting antibodies to the spike SARS-CoV-2 protein are non-neutralizing and have no effect on virus replication control [42]. Moreover, reinfection with CoVs is common, and the levels of cross-reactive antibodies and cross-reactive T cells increase with age, becoming higher in adults than in children [43]. Finally, children with COVID-19 have antibody levels to common CoVs similar to those of healthy children [44].

On the other hand, pre-existing immunity against common CoVs may be negative and lead to a more severe COVID-19 outlook. Non-neutralizing antibodies may form complexes with SARS-CoV-2, which can more easily infect cells and favor the development of antibody-dependent enhancement (ADE) of virus infection, one of the mechanisms considered at the base of MIS-C [45].

## 5. Previous Enhanced Activation of the Innate Immune System

During the first years of life, the immune system of children is continuously stimulated. The incidence of infections, particularly viral respiratory infections, is extremely common. Even otherwise healthy children suffer from up to 6–8 episodes each year [46]. Moreover, according to the recommended immunization schedule [47], children receive several doses of many vaccines specifically administered to prevent some of the most common and sometimes severe infectious diseases. For years, it was thought that this strong immune stimulation did not influence innate immune system activity. Only recently, it has been evidenced that the ability of innate immunity to handle pathogens can be modulated by previous immune stimulations [48]. Macrophage, NK cell and PRR activity can be modified and reprogrammed by epigenetic stimuli. Innate antigen-presenting cells are more rapidly activated, proinflammatory cytokines are more rapidly released and more rapid clearance of pathogens can occur [49]. This means that after a first response to an infectious agent, the innate immune system can remain activated and respond differently when exposed to a second stimulation by the same or a different agent. Trained programs of innate immunity vary with the characteristics of the infectious agent; although reduced activity can be generated in some cases, innate immune memory leads to a stronger response than initially evidenced. The duration of innate immune system memory is shorter than that of adaptive immunity. Generally, it lasts a few months, although in the case of vaccines, it has been thought that it could last up to five years [50].

The clinical importance of modulation of the innate immune system is evidenced by the protective effect against infections due to different pathogens in children previously immunized with vaccines. Recent randomized trials have shown that children who have received the Bacillus Calmette–Guerin (BCG) vaccine, measles vaccine, smallpox vaccine and oral polio vaccine had a substantial reduction in morbidity and mortality in the following months, which could not be explained by prevention of the target disease [51,52,53]. Similar stimulation of the innate immune system with a reduced incidence of new infectious episodes can also be supposed for children who previously suffered from repeated infectious episodes when they receive adequate immune stimulation. This hypothesis is supported by evidence that oral administration of the product of alkaline lysis of 21 strains of common bacterial respiratory tract pathogens, mimicking bacterial respiratory infections, is associated with dendritic cell activation and the release of chemokines that act on monocytes and NK cells, as well as prophagocytic chemokines that induce polymorphonuclear neutrophil migration and the release of antimicrobial peptides [54,55,56].

However, the real importance of modulation of innate immune system activity for protection of children from further infections is unknown, particularly in the case of SARS-CoV-2 infection. As already reported, children with COVID-19 show reduced serum cytokine levels and this contrasts with the hypothesis of an enhanced activation of the innate immune system. Moreover, results of studies have suggested that the BCG vaccine could provide protection against SARS-CoV-2 infection [57], and the use of a bacterial lysate can reduce the incidence, prevalence and/or duration of further infections in children with a history of recurrent respiratory tract infections [58,59]. Regarding the role of the BCG vaccine, it has been shown that the severity of COVID-19 is significantly lower in countries with universal BCG vaccination policies than in those without [60,61]. However, analysis of the available data has clearly highlighted that no definitive conclusions in this regard can be drawn, as the studies were not systematically corrected for confounding variables, such as cardiovascular death rates and smoking prevalence. When confounding variables were considered, no correlation between overall BCG vaccination policy and spread of SARS-CoV-2 and its associated mortality was found [62]. Further studies are ongoing. However, they are only enrolling adults and will not explain the importance of innate immune stimulation in lowering the severity of COVID-19 in children [62].

## 6. Other Factors

### 6.1. Exposure to SARS-CoV-2 and the Presence of Underlying Disease

In most countries, schools were closed during the pandemic, even when adults were allowed to travel and work. From this, it was deduced that children might have had less intense exposure to SARS-CoV-2. Lower exposure could have led to a reduced risk of diffusion, and some epidemiological studies have supported this hypothesis, showing that children were generally infected by adults, as they rarely presented as index cases in household clusters [63,64,65]. Finally, secondary infections in the COVID-19 pandemic were characterized by reduced pathogenicity, as also evidenced during the SARS-CoV and MERS-CoV epidemics [66]. If children were predominantly infected by adults and all the secondary cases were less severe, these findings could explain why most children with SARS-CoV-2 infection are asymptomatic and why COVID-19 is generally milder in children than in adults. Unfortunately, virological data collected in children with SARS-CoV-2 infection do not support lower exposure as a reason for mild COVID-19 in children. Not all the studies confirmed that viral load in infected children differed between asymptomatic and symptomatic patients, and not all the studies indicated that viral load was lower in symptomatic children than in adult symptomatic patients [67,68,69].

In adults, the presence of underlying disease is strongly associated with an increased risk of severe COVID-19. Underlying medical conditions clearly associated with an increased risk of hospitalization, admission to the intensive care unit (ICU) and death include cancer; chronic kidney disease; chronic obstructive pulmonary disease; heart conditions, such as heart failure, coronary artery disease or cardiomyopathies; weakened immune system from solid organ transplant; obesity; sickle cell disease; and type 2 diabetes mellitus [70]. The incidence of these diseases in children is very low, and this could be a protective factor. However, available data on the role of underlying disease in children remain questionable. The role of prehospital comorbidities as a factor for hospitalization and the severe course of COVID-19 in children has not been defined. Together with studies showing that most children admitted to the PICU were suffering from a severe underlying disease [71,72], other studies reported no difference in the severity of COVID-19 among children with underlying comorbidities and healthy children [73,74].

### 6.2. Melatonin

Several studies have shown that melatonin has anti-inflammatory and antioxidative properties. Moreover, it can regulate immune responses. Administration of melatonin improves the proliferation and maturation of NK cells, T and B lymphocytes, granulocytes and monocytes [75] and, in the acute phase of inflammation, can reduce the level of proinflammatory cytokines [76,77]. All these activities can play a relevant role in reducing clinical manifestations due to viruses and bacteria, as repeatedly reported in animal models [78]. In SARS-CoV-2-infected patients, altered immune system function, excessive inflammation, and an activated cytokine storm are the basis for the clinical manifestations of the disease [79]. The potential ability of melatonin to contain these problems has led to the conclusion that melatonin could be used in the prevention and treatment of COVID-19. A randomized trial in this regard is ongoing. Melatonin secretion is strictly age-related, with infants having the highest concentration. This could be one of the explanations for the lower severity of COVID-19 in children. However, due to the lack of studies in this regard, no definitive conclusions can be drawn.

### 6.3. Coagulation Abnormalities

Coagulation abnormalities that lead to venous and arterial thromboembolic complications and disseminated intravascular coagulation can be observed in a substantial number of adult patients with severe COVID-19 [80]. Excess thrombin generation and fibrinolysis shutdown induced by viral infection and the subsequent endothelial cell dysfunction are considered the most important causes of this complication [81,82]. Hypoxia due to lung lesions can further worsen coagulation abnormalities [83].

Children have important physiologic differences in the hemostatic system compared to adults, leading to longer bleeding times during the first 10 years of life [84]. The concentration of the majority of blood-clotting proteins is significantly lower in the first periods of life and increases with age. Moreover, concentrations of inhibitors can vary. In particular, it was reported that the mean values of seven coagulant factors (II, V, VII, IX, X, XI, XII) were significantly lower than adult values, while those of inhibitors, such as alpha 2-macroglobulin and protein C1 inhibitors, were elevated [85]. These differences explain why children are more protected from thrombosis than adults, as epidemiologic reports seem to indicate. Children with congenital deficiencies of AT, protein C or protein S do not suffer from thrombosis until adolescence [86,87,88]. Contrary to adults, in children undergoing surgical procedures, the risk of secondary thrombosis is uncommon [89]. Starting from these premises, it has been supposed that developmental hemostasis could provide a protective mechanism against the development of severe COVID-19 in children. However, no definitive data in this regard are presently available.

### 6.4. Vitamin D

Vitamin D has many mechanisms by which it reduces the risk of microbial infection and death, including physical barrier, cellular natural immunity and adaptive immunity [90]. There is evidence on the role of vitamin D in regulating the immune response to viral infections, and data from most observational studies confirm an association between lower vitamin D levels and increased susceptibility to respiratory infections [90].

Interestingly, some studies showed a high frequency of vitamin D insufficiency in COVID-19 patients admitted to intensive care units [91]. Moreover, some pilot studies demonstrated that administration of a high dose of 25-hydroxyvitamin D significantly reduced the need for intensive care unit treatment of patients requiring hospitalization due to proven COVID-19 [91]. Considering data overall, the effects of vitamin D supplementation during COVID-19 infection remain controversial. Looking ahead, clinical studies are needed to define better cut offs for vitamin D levels and, finally, which dosage is the best.

## 7. Conclusions

Although most children are clearly protected from the development of severe COVID-19, the reasons for this peculiarity are not fully understood. Several factors can be called into question, but none are completely convincing. Developmental variations in immune system function together with the potential role of repeated antigen stimulation in the first periods of life on innate immunity are more widely studied. As the few children who develop the most severe form of pediatric COVID-19, MIS-C, have certain alterations in the immune system response to SARS-CoV-2 infection, studies about the relationships between SARS-CoV-2 and the immune system of the host are essential to understand the reasons for the age-related differences in the severity of COVID-19. On the other hand, comparisons of adults’ and children’s response to infectious diseases may be intriguing and the object of further investigations.

## Data Availability

Not applicable.
